# Serine-arginine protein kinase 1 (SRPK1) is elevated in gastric cancer and plays oncogenic functions

**DOI:** 10.18632/oncotarget.18734

**Published:** 2017-06-28

**Authors:** Xiaotao Xu, Yuehua Wei, Shidong Wang, Man Luo, Heng Zeng

**Affiliations:** ^1^ Department of Oncology, Renmin Hospital, Wuhan University, Wuhan, Hubei, 430060, China; ^2^ Department of Orthopedics, Tongji Hospital, Tongji Medical College, Huazhong University of Science and Technology, Wuhan, Hubei, 430030, China

**Keywords:** gastric adenocarcinoma, invasion, prognosis, proliferation, SRPK1

## Abstract

Serine-arginine protein kinase 1 (SRPK1) phosphorylates proteins involved in the regulation of several mRNA processing pathways including alternative splicing. SRPK1 has been reported to be over-expressed in multiple cancers including prostate, breast, lung and glioma. Several studies further identified that inhibition of SRPK1 showed tumor-suppressive effects, thus raising SRPK1 as a novel candidate chemotherapy target. Interestingly, SRPK1 plays tumor suppressing role in mouse embryonic fibroblasts, on that SRPK1-silencing induces cell transformation. Therefore, the effect of SRPK1 seems heterogeneously in different cell types and tissues. The existence and role of SRPK1 in gastric cancer (GC) hasn’t been reported. Here we investigated the expression pattern of SRPK1 in GC by immunohistochemistry and found that it was up-regulated in tumor tissues, where its expression was correlated with tumor grade and prognosis. Further, we explored the signaling mechanism of SRPK1 in promoting GC progression, which revealed that both PP2A and DUSP6 phosphatases impaired the oncogenic effects of SRPK1. However, we didn’t find any direct interaction between SRPK1 with PP2A or DUSP6, indicating PP2A and DUSP6 function by regulating the downstream effectors of SRPK1. Our study not only revealed the clinical significance of SRPK1 in GC, but also provided new evidence for its signaling modulation which is invaluable for novel chemotherapy development.

## INTRODUCTION

Gastric cancer (GC) is the one of the most common carcinoma and the second leading cause of cancer-related death worldwide [1, 2]. The only potentially curative treatment for GC is R0 surgical resection [3]. However, most GC is diagnosed at an advanced stage [4], which is the major cause of unsatisfied overall survival (OS). In addition, more than 50% of patients will experience disease recurrence after surgery [5]. Therefore, despite advances in surgical intervention and chemotherapy, the overall prognosis of patients with advanced GC remains poor [6]. Thus, it is still an urgent need to identify novel biomarkers which correlate with GC tumorigenesis and progression.

Serine/arginine-rich domain proteins (SR proteins) mainly consist of the splicing factors for small nuclear ribonucleoprotein particles (snRNPs) and non-snRNP that concentrate in 'speckles' in the nucleus of interphase cells. SR protein-specific kinase-1 (SRPK1) was firstly identified in 1994 and can phosphorylate one of the SR proteins, namely SC35 [7]. The association of phosphorylation of SR proteins with SRPK1 activity, together with the fact that SRPK1 was the only major kinase for SR proteins obtained during purification, strongly suggests that SRPK1 is responsible for phosphorylation of SR proteins during the cell cycle *in vivo* [8–10]. Also, SRPK1 is highly expressed in testis, revealing its role in regulating cell division [11].

SRPK1 as well as its downstream targets have been shown to be involved in numerous biological and pathological processes. Dysregulation of SRPK1 has been reported in several cancer types, such as colon, breast, prostate, pancreas and lung cancer [12–15]. High expression of SRPK1 can induce hyper-phosphorylation of SRSF1 protein [16], subsequently increase the transcription and translation of certain proteins, including VEGFs, rising up its role as an oncogenic protein. However, a recent study reported that ablation of SRPK1 in mouse embryonic fibroblasts induced cell transformation and promote tumorigenesis [17]. Therefore, the role of SRPK1 seems distinct in different cell types.

Here in this study, we firstly explored the expression level of SRPK1 in clinical GC tissues. Statistical analyzes revealed that SRPK1 high expression was correlated with advanced tumor stage and poor prognosis. The clinical results promoted us to further investigate the underlying mechanisms of SRPK1 in regulating GC progression. Cellular experiments showed that SRPK1 can enhance cell proliferation and invasion via AKT and ERK signaling pathways, whereas PP2A (protein phosphatase 2A) and DUSP6 (dual specificity phosphatase 6) both attenuated its oncogenic effects.

## RESULTS

### Patients characteristics

For the 158 cases enrolled in this study, 105 cases (66.5%) were male. Most of the tumor localization was in the gastric body (67/158, 42.4%) or pylorus (61/158, 38.6%). At the time of tumor resection, the largest tumor diameter was less than 5.0 cm in 91 patients (57.6%), and the tumor already invaded into the subserosa or serosa layer (tumor invasion depth as T3-T4) in 104 cases (65.8%). Nighty-five patients (60.1%) were classified as TNM stage III-IV at the time of diagnosis. The serious invasion depth and advanced TNM stage mainly resulted from the unobvious disease phenomenon at early stage. The detailed patients’ information was provided in Table 1.

**Table 1:** Overview for the characteristics of the cohort

Clinicopathologic features	Case number	Percentage
Age		
≤55 ys	61	38.6%
>55 ys	97	61.4%
Gender		
Female	53	33.5%
Male	105	66.5%
Localization		
Upper	30	19.0%
Middle	67	42.4%
Lower	61	38.6%
Tumor size		
≤5.0 cm	91	57.6%
>5.0 cm	67	42.4%
Invasion depth		
T1-T2	54	34.2%
T3-T4	104	65.8%
Differentiation		
Well	15	9.5%
Modern	74	46.8%
Poor	69	43.7%
TNM stage		
I-II	63	39.9%
III-IV	95	60.1%
SRPK1 expression		
Low	75	47.5%
High	83	52.5%

### High SRPK1 expression was correlated with advanced tumor stage

**Figure 1:**
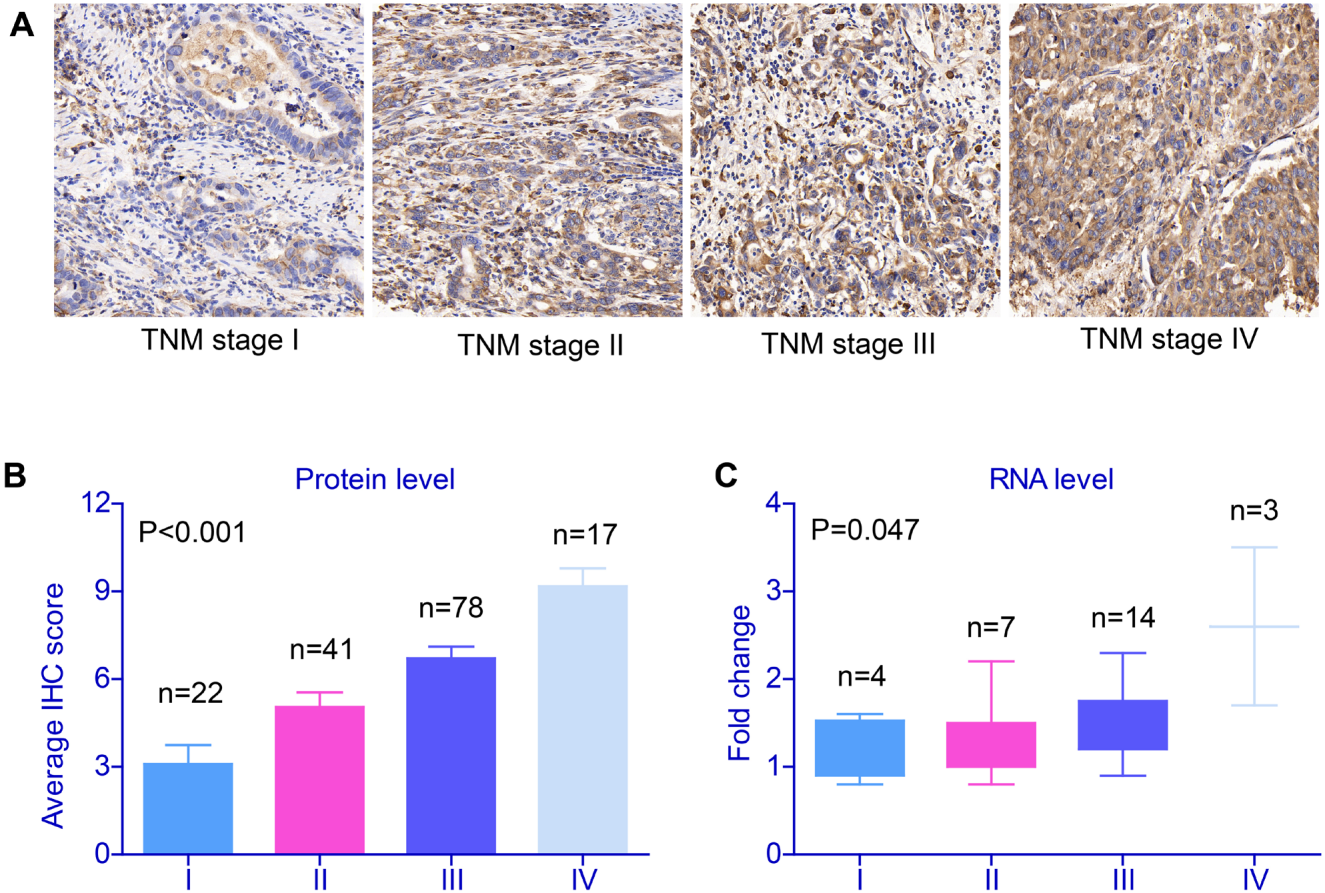
Validation of SRPK1 expression in gastric cancer tissues **(A)** SRPK1 protein expression as determined in an IHC assay of tissues from patients with gastric adenocarcinoma. Among 158 patients, tissues from 83 showed high SRPK1 staining, mainly in the cytoplasm and partly in the nucleus of cancer cells (IHC score≥8). Moreover, both the protein **(B)** and RNA **(C)** levels of SRPK1 in gastric adenocarcinoma tissues were positively correlated with tumor stage (P<0.001 and P=0.047, respectively). Data was from three independent experiments and statistical analyzes were conducted with One-way ANOVA test, P value was showed correspondingly.

SRPK1 expression was localized to the cytoplasm (Figure 1A) in GC tissues, and 52.5% (83/158) of the cases were categorized as high expression, while 47.5% (75/158) were grouped as low expression. Importantly, the protein expression level of SRPK1 was positively associated with the tumor stage (P<0.001, Figure 1B, Table 2). Therefore, we further collected 28 pairs of fresh-frozen tissues, among them, 4 cases were diagnosed with TNM stage I, 7 cases with stage II, 14 cases with stage III, and the other 3 cases with stage IV. By conducting RT-qPCR assay, we found that the mRNA level of SRPK1 was also correlated with TNM stages (P=0.047, Figure 1C), which was consistent with the protein levels.

**Table 2:** Correlations between SRPK1 expression with clinicopathological factors

Clinicopathologic features	SRPK1 expression level		Chi-square P value1
	Low (n=75)	High (n=83)	
Age			
≤55 ys	30	31	0.733
>55 ys	45	52	
Gender			
Female	30	23	0.102
Male	45	60	
Localization			
Upper	11	19	0.414
Middle	33	34	
Lower	31	30	
Tumor size			
≤5.0 cm	48	43	0.121
>5.0 cm	27	40	
Invasion depth			
T1-T2	35	19	0.002*
T3-T4	40	64	
Differentiation			
Well	8	7	0.658
Modern	37	37	
Poor	30	39	
TNM stage			
I-II	44	19	<0.001*
III-IV	31	64	

### SRPK1 was an unfavorable biomarker for the prognosis of GC patients

**Figure 2:**
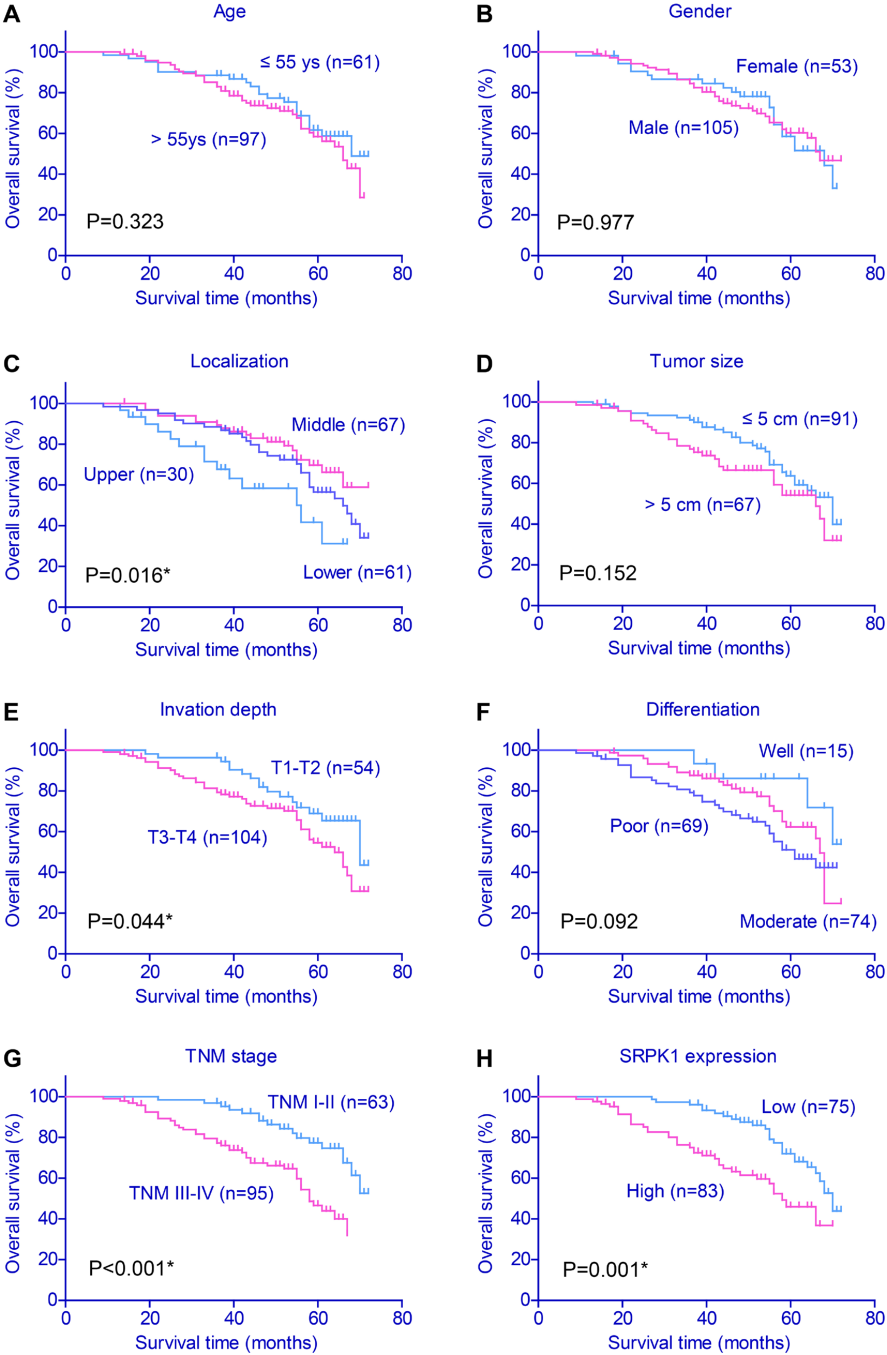
Kaplan–Meier survival plots of gastric cancer patients The effects of various clinicopathological variables on prognosis were assessed by Kaplan–Meier survival analysis and log-rank test. Tumor location (P=0.016), invasion depth (P=0.044), TNM stage (P<0.001) and SRPK1 expression (P=0.001) were all associated with the clinical outcomes of gastric cancer patients.

The overall 5-year survival (5-year OS) of the cohort was 59.6%, and the median survival time was 53.0 months. Kaplan–Meier plots (Figure 2) showed that patients with cardia tumor location showed poorer prognosis (P=0.016). Conventional prognostic factors also included tumor invasion depth (P=0.044) and TNM stage (P<0.001). Interestingly, high expression of SRPK1 also indicated unfavorable clinical outcomes (P=0.001, Table 3). In contrast, patients’ age, gender, tumor size or tumor differentiation showed no statistical significance.

**Table 3:** Kaplan-Meier univariate survival analysis for GC

Clinicopathologic features	5-year OS (%)	OS months (Mean ± S.D.)	Univariate P value
Age			
≤55 ys	61.6%	59.7 ± 2.3	0.323
>55 ys	58.4%	57.1 ± 1.8	
Gender			
Female	58.5%	58.1 ± 2.4	0.977
Male	60.3%	58.5 ± 1.8	
Localization			
Upper	41.7%	48.4 ± 3.6	0.016*
Middle	69.7%	61.9 ± 2.0	
Lower	56.5%	58.5 ± 2.2	
Tumor size			
≤5.0 cm	63.6%	60.6 ± 1.7	0.152
>5.0 cm	54.2%	55.1 ± 2.5	
Invasion depth			
T1-T2	68.9%	62.5 ± 2.0	0.044*
T3-T4	54.5%	55.9 ± 1.9	
Differentiation			
Well	86.2%	66.0 ± 3.0	0.092
Modern	62.3%	59.8 ± 2.0	
Poor	51.1%	54.3 ± 2.4	
TNM stage			
I-II	77.3%	64.8 ± 1.6	<0.001*
III-IV	46.6%	52.2 ± 1.9	
SRPK1 expression			
Low	72.0%	63.9 ± 1.4	0.001*
High	46.0%	52.2 ± 2.3	

Abbreviations: GC, gastric cancer; OS, overall survival; SRPK1, serine-arginine protein kinase 1.

The Kaplan–Meier survival results promoted us to further investigate whether SRPK1 can serve as an independent prognostic biomarker. Cox regression hazard analysis demonstrated that both advanced TNM stage (P=0.014) and high SRPK1 expression (P=0.035, Table 4) were significant risk factors for the OS of GC patients.

**Table 4:** Multivariate analysis for hazard factors of GC survival

Variables	HR (95% CI)	Multivariate P value
Localization		
Middle or lower	0.800 (0.545-1.176)	0.256
Upper	Reference	
Invasion depth		
T1-T2	0.848 (0.413-1.743)	0.654
T3-T4	Reference	
TNM stage		
III-IV	2.609 (1.218-5.590)	0.014*
I-II	Reference	
SRPK1 expression		
High	1.818 (1.044-3.165)	0.035*
Low	Reference	

Abbreviations: GC, gastric cancer; HR, hazard ratio; SRPK1, serine-arginine protein kinase 1.

### SRPK1 promoted proliferation and invasion of GC cells

**Figure 3:**
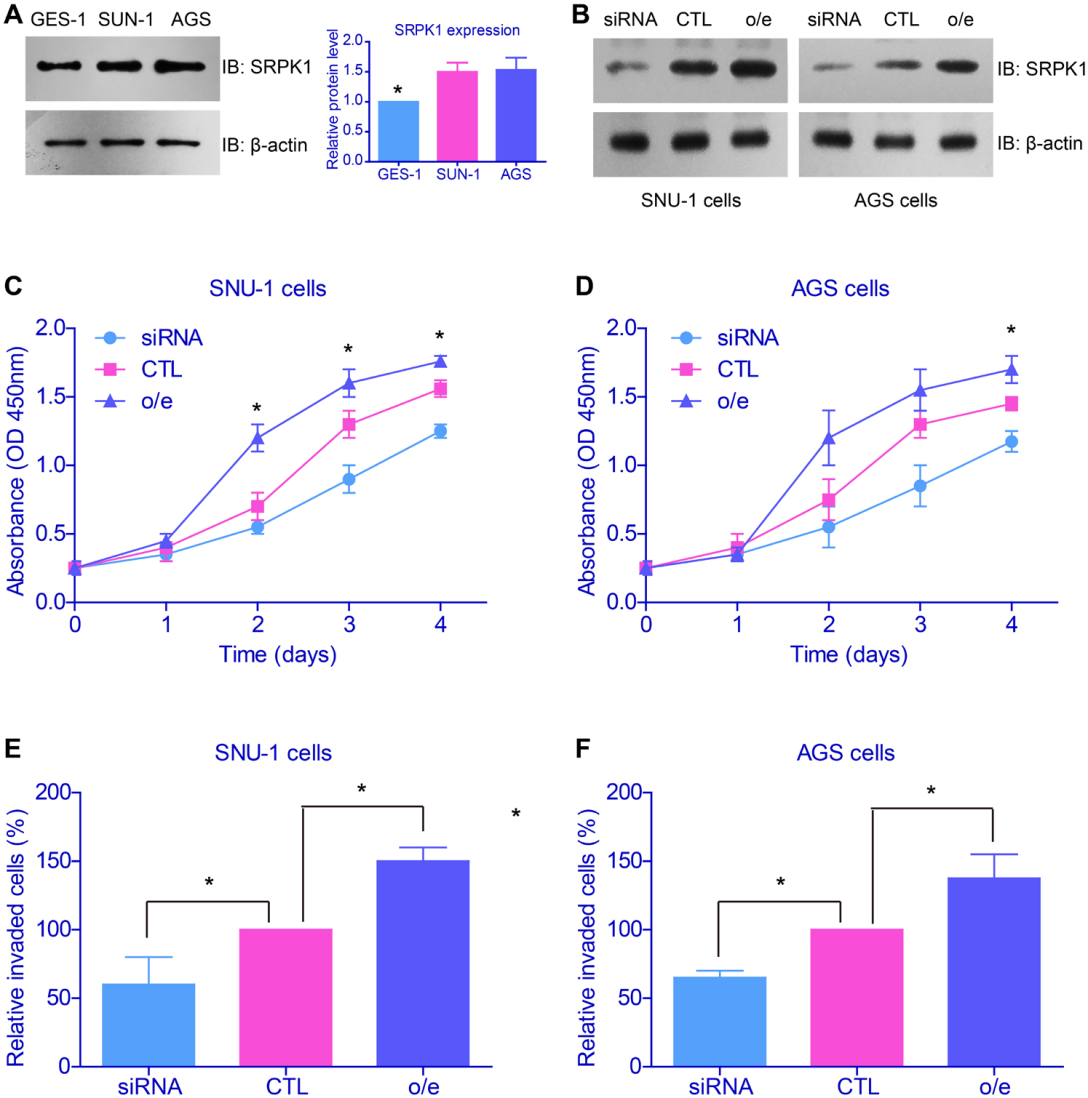
SRPK1 promoted proliferation and invasion of gastric cancer cell lines **(A)** Western blot results showed that SRPK1 expression was higher in gastric adenocarcinoma cells (SUN-1 and AGS cells) than normal gastric epithelial cells (GES-1 cells). **(B)** Transfection efficiency of SRPK1 overexpression (o/e) and knockdown in both SUN-1 and AGS cells were confirmed. The proliferation **(C, D)** and invasion **(E, F)** capacities of tumor cells were tested by CCK-8 and Matrigel-Transwell assays, respectively. Both the cell viability and invasion were up-regulated upon SRPK1 overexpression, whereas SRPK1-siRNA impaired the oncogenic characteristics.

The clinical results indicated the potential oncogenic role of SRPK1 in GC, and next we performed cellular studies to verify its detailed functions and mechanisms. Western blot results showed that SRPK1 expression was higher in gastric adenocarcinoma cells (SUN-1 and AGS cells) than normal gastric epithelial cells (GES-1 cells, Figure 3A). Then we conducted overexpression and knockdown of SRPK1 in both SUN-1 and AGS cells (Figure 3B). The proliferation and invasion capacities of tumor cells were tested by CCK-8 and Matrigel-Transwell assays, respectively. As expected, both the cell viability and invasion were up-regulated upon SRPK1 overexpression, whereas SRPK1-siRNA impaired the oncogenic characteristics (Figures 3C-3F).

### AKT and ERK were downstream effectors of SRPK1 in tumorigenesis

**Figure 4:**
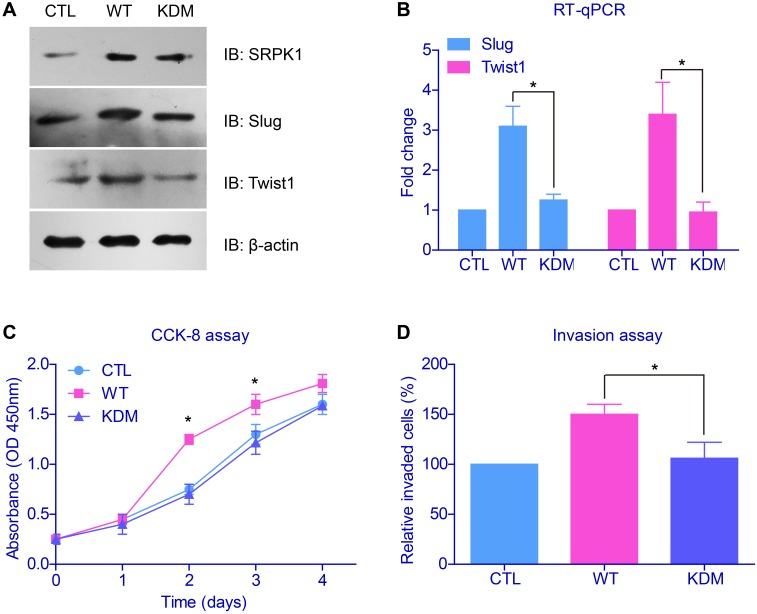
Catalytic activity of SRPK1 was critical in promoting tumor progression **(A)** Immunoblotting results revealed that wild type SRPK1 (WT) overexpression increased the protein levels of epithelial-mesenchymal transition (EMT) biomarkers. However, transfection of the kinase-dead mutant of SRPK1 (KDM) showed no significant oncogenic alteration on Slug and Twist1. **(B)** The mRNA levels of EMT markers were tested by RT-qPCR and showed comparable results with protein levels. Overexpression of SRPK1-KDM neither showed effect on cell proliferation **(C)** nor invasion **(D)**, compared with control groups.

Taking into consideration that SRPK1 was an important kinase, we then wanted to test whether its kinetic activity was indispensable in tumor promoting. We generated the kinase-dead mutant (KDM) of SRPK1 by mutating the critical catalytic lysine of amino acid 109 into alanine [18]. Immunoblotting results revealed that SRPK1 overexpression increased the mRNA and protein levels of epithelial-mesenchymal transition (EMT) biomarkers (Figures 4A, 4B). Moreover, IHC results implicated significant correlation between SRPK1 and twist1 (Supplementary Figure 1). On the other hand, the SRPK1-KDM showed no significant oncogenic effects on Slug and Twist1, two of the most important EMT markers. Consistently, transfection of SRPK1-KDM neither exhibited changes on proliferation nor invasion profiles, compared to control cells (Figures 4C, 4D).

**Figure 5:**
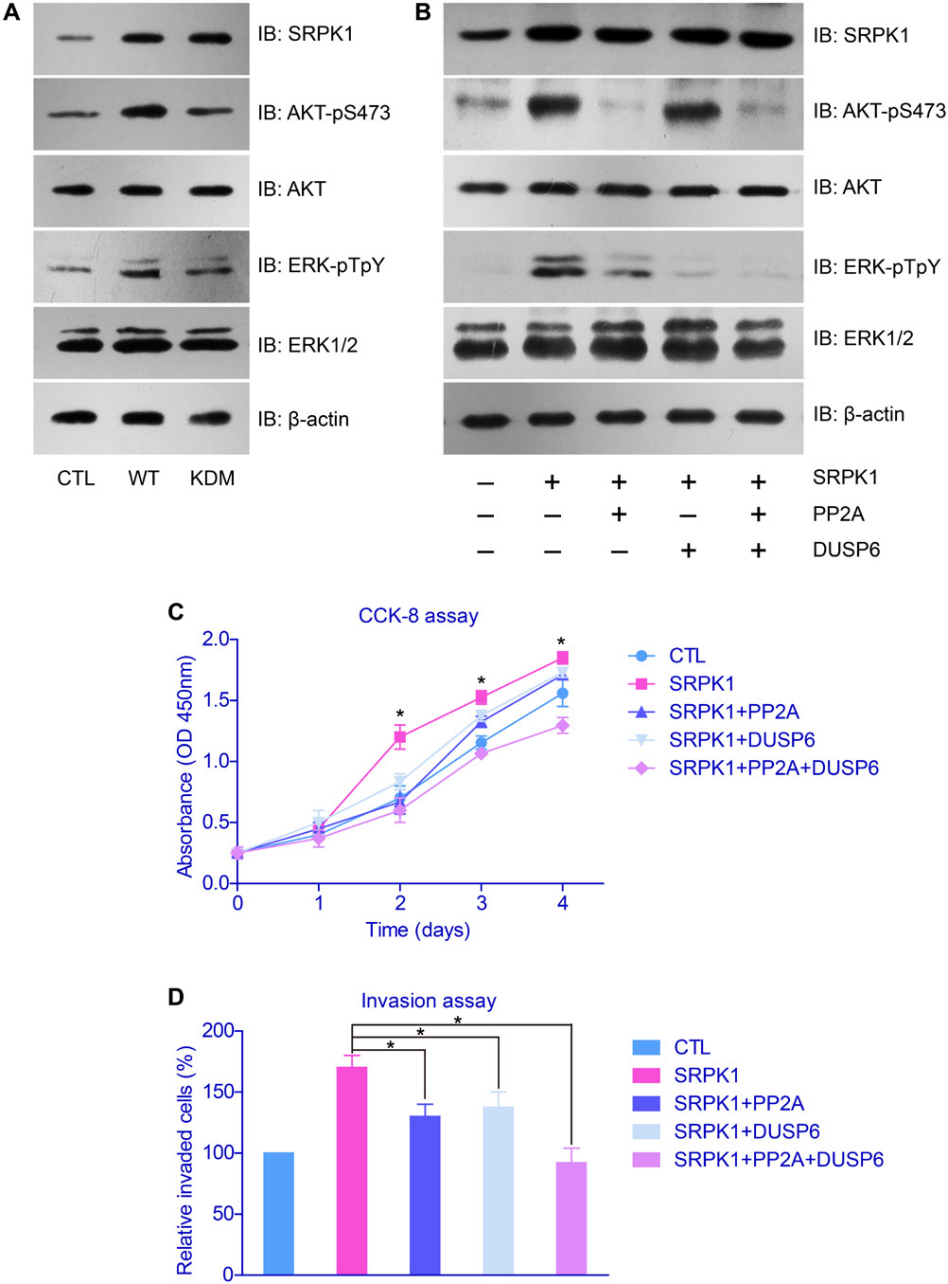
AKT and ERK were down-stream effectors of SRPK1 in gastric cancer cells **(A)** SRPK1-overexpression significantly elevated the phosphorylation levels of AKT and ERK proteins, whereas SRPK1-KMD showed little effect. **(B)** The activation function of AKT and ERK by SRPK1 can be impaired upon overexpression of PP2A and DUSP6, respectively. The facilitated effects of SRPK1 on cell proliferation **(C)** and invasion **(D)** were also down-regulated by PP2A and DUSP6, indicating the critical role of AKT and ERK in SRPK1-mediated tumor progression.

After we confirmed that the oncogenic role of SRPK1 was functioned by its catalytic activity, we then explored its down-stream effectors. Cellular studies showed that both the phosphorylation of AKT and ERK were enhanced upon SRPK1-overexpression, whereas no significant change with SRPK1-KDM transfection (Figure 5A). Therefore, SRPK1 can phosphorylate and activate AKT and ERK, both are critical in controlling cell proliferation and invasion [19].

### PP2A and DUSP6 attenuated the oncogenic effects of SRPK1 with indirect manners

To study the functional mechanisms of SRPK1 in promoting GC progression, we further tested the signaling network on AKT and ERK phosphorylation. It has been reported that PP2A was a phosphatase toward phosphor-AKT [20] and DUSP6 was a phosphatase for phosphor-ERK [21]. By transfected cells with PP2A and/or DUSP6, we found that the activation of AKT and ERK were inhibited, correspondingly (Figure 5B). In addition, the proliferation and invasion capacities of tumor cells were significantly down-regulated after overexpressed PP2A and/or DUSP6, compared with control groups (Figures 5C, 5D).

**Figure 6:**
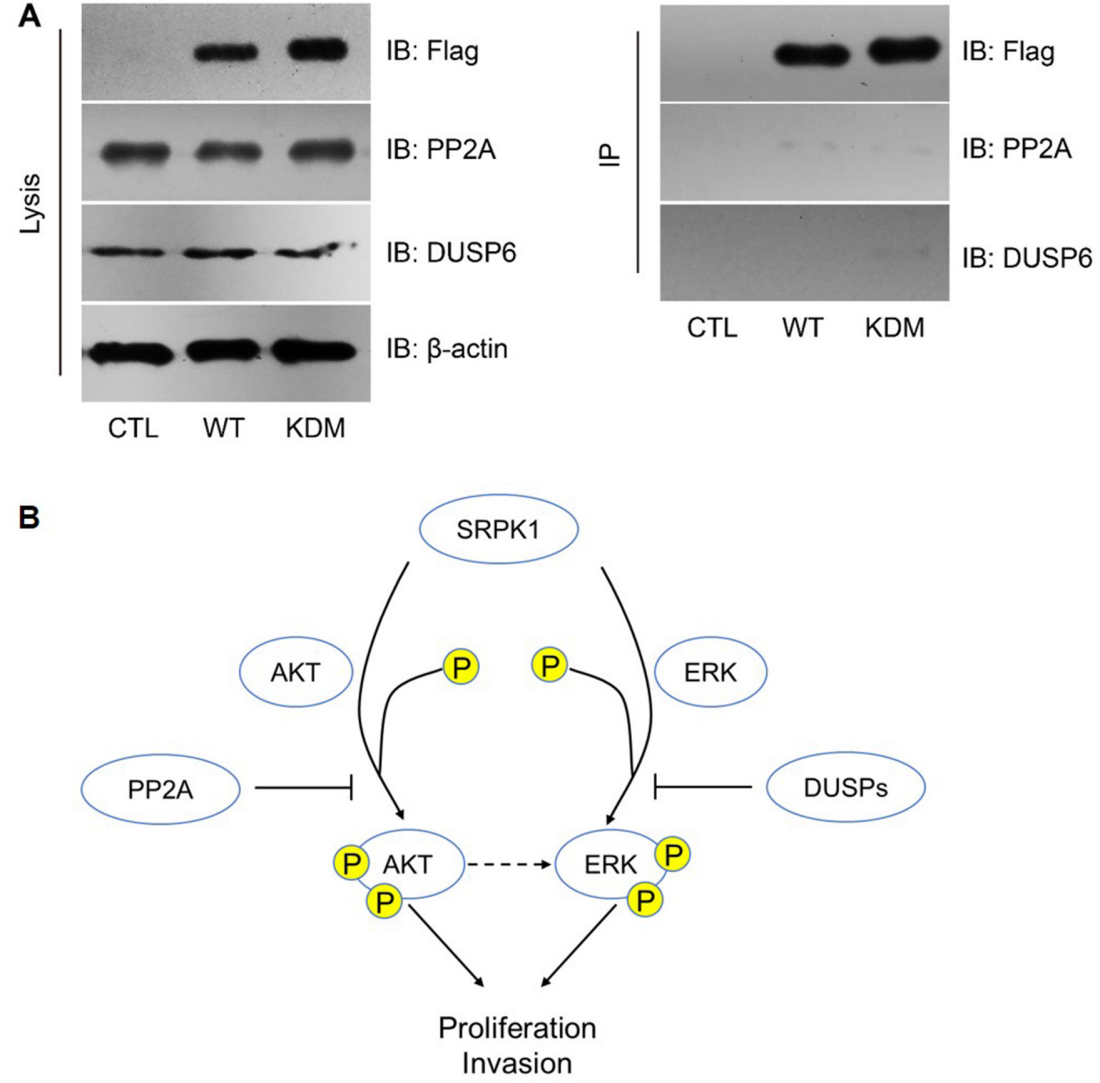
PP2A and DUSP6 inhibited SRPK1 signaling by indirect manners **(A)** Neither PP2A nor DUSP6 was interacted with SRPK1, as reflected by immunoprecipitation (IP) assay. **(B)** Schematically model of the functional network involving SRPK1, AKT, ERK, PP2A and DUSP6.

However, we didn’t find any interaction between SRPK1 with PP2A or DUSP6 (Figure 6A), indicating that PP2A and DUSP6 may regulate down-stream effectors of SRPK1 rather than SRPK1 itself (Figure 6B).

## DISCUSSION

SRPK1 is a protein kinase that specifically phosphorylates proteins containing serine–arginine-rich (SR) domains [22]. SR proteins are involved in regulating several RNA-processing pathways, including RNA stability, alternative splicing, and translation [23]. Both SRPK1 and its downstream targets have been shown to be involved in a number of biological and pathological processes [24]. Although there have been several studies reporting the dysregulation of SRPK1 in malignancies [25], there was no evidence about its role in GC.

Our study detected the expression of SRPK1 in GC for the first time, and demonstrated that SRPK1 was up-regulated in GC cells and tissues. Immunostaining analysis showed that the expression level of SRPK1 protein in histological sections was significantly correlated with clinical characteristics and reduced survival time of gastric cancer patients. Multivariate analysis revealed that SRPK1 expression might be an independent prognostic indicator of survival in GC patients. Taken together, our study suggests that SRPK1 is a novel marker for the prognosis of GC. Furthermore, ectopic overexpression of SRPK1 promoted, while silencing expression of SRPK1 inhibited, the proliferation and invasion of GC cells. In addition, we found that AKT and ERK are involved in the SRPK1 signaling pathway, which was interdicted by PP2A and DUSP6 phosphatases.

Interestingly, although our study revealed that SRPK1 can phosphorylate AKT, AKT was able to phosphorylate SRPK1 reciprocally [26]. However, we didn’t test the phosphorylation levels of SRPK1 due to lacking specific antibodies. Analogically, ERK may also regulate the phosphorylation and functions of SRPK1, but this will need more evidence to verify. Besides ERK and AKT, SRPK1 can activate several other tumor-related proteins in distinct tumor types, such as JNK [27] and VEGFs [28–30]. Certain up-stream regulators for SRPK1 were also identified, including RhoA [31], Wnt [15], TGF-β [27], long non-coding RNAs [25] and human papillomavirus type 1 E1^E4 protein [32].

The complicated signaling network of SRPK1 indicates its invaluable potential in drug development. Indeed, a recent study reported highly potent, selective, and cell active SRPK1 inhibitors, which also have antiangiogenic properties *in vivo* [33], shedding light on its novel anti-tumor applications.

## CONCLUSION

SRPK1 is an oncogenic protein in GC, its high expression is correlated with advanced TNM stage and poor prognosis. SRPK1 functions via AKT and ERK signaling pathway, whereas PP2A and DUSP6 show antagonistic effects.

## MATERIALS AND METHODS

### Patients and samples

A total of 158 patients was retrospectively included in this study. All patients were diagnosed as gastric adenocarcinoma by pathological examinations, and all the patients underwent R0 surgical resections in Renmin Hospital Affiliated to Wuhan University from March 2000 to March 2002. During the study, we also collected 28 pairs of tumor tissues with adjacent normal tissues, and were frozen in liquid nitrogen until analysis.

### Immunohistochemistry staining (IHC) and IHC evaluation

One hundred fifty-eight formalin-fixed, paraffin-embedded GC tissues were used for SRPK1 IHC experiments. After deparaffined and blocking, antigen-antibody reaction was performed at 4°C overnight. The 3,3′-diaminobenzidine (DAB) reagents were applied to determine the signal from the immune-reaction. All slides were counterstained with hematoxylin. The primary SRPK1 (Abcam, Cambridge, MA, USA; ab58002; dilution 1:100) antibody was used for the immunostaining. Primary antibody was replaced with the phosphate-buffered saline (PBS) as a negative control.

The immunostaining results were assigned by two independent pathologists based on both the staining intensity and proportion of positive cells. The percentage score was calculated based on the proportion of positive cells (0, none; 1, ≤25 %; 2, 26–50 %; 3, 51–75 %; 4, >75 %). The intensity score was evaluated according to the positive intensity (0, none; 1, weak; 2, moderate; 3, strong). The total IHC score for SRPK1 expression, ranging from 0 to 12, was the product of the percentage and intensity scores. The protein expression of SRPK1 was categorized as low (IHC score 1–7) or high (IHC score 8–12) for subsequent statistical analysis.

### RNA isolation and reverse transcription quantitively polymerase chain reaction (RT-qPCR)

Total RNA from cultured cells and GC tissues was isolated using the RecoverAll Total Nucleic Acid Isolation Kit (Ambion) according to the manufacturer’s instruction. The cDNA was then synthesized from total RNA using the SuperScript II Reverse Trancriptase (Invitrogen). RT-qPCR was performed with the Applied Biosystems 7500 Sequence Detection system, using iQ™ SYBR Green Supermix (BioRad Laboratories, Hercules, CA, USA). The data was normalized to GAPDH housekeeping gene and calculated as 2^−ΔΔCT^ method [34]. Sequences of the primers for RT-qPCR are shown below:

SRPK1: forward primer, 5′-TAATGATTAT TGTAAAGGAG-3′; and reverse primer, 5′-GAACAACC ATTTCTCTATTT-3′.
Slug: forward primer, 5'-CTTCCTGGTCAAGA AGCA-3'; and reverse primer, 5'-GGGAAATAATCACT GTATGTGTG-3'.
Twist1: forward primer, 5'-GGAGTCCGCAG TCTTACGAG-3'; and reverse primer, 5'-CCAGCTTGAG GGTCTGAATC-3'.
GAPDH: forward prime, 5′-ACATCCCCTCAC CAATAACAAC-3′; and reverse primer: 5′-TAGCCAAAT CATACTGCTCGTC-3′.

### Cell culture and transfection

Normal human gastric epithelial cells (GES-1 cells) were purchased from American Type Culture Collection (ATCC, Manassas, VA, USA). Human gastric adenocarcinoma cell lines, SNU-1 cell line and AGS cell line, were also obtained from ATCC. All cells were cultured in DMEM (Life Technologies, Grand Island, NY, USA) supplemented with 10% fetal bovine serum (FBS, Life Technologies, Grand Island, NY, USA) and 1% penicillin/streptomycin (Life Technologies) at 37°C with 5% CO2.

Homo sapiens *SRPK1* cDNA in pDONR223 vector was purchased from Addgene (Addgene plasmid 23582) [35], a Flag-tag was further added into the construct by PCR and confirmed by DNA sequencing. The siRNA sequences for SRPK1 was 5'-GATCATCAAATCCAATTA-3’ [36] and synthesized by GenePharma (Shanghai, China).

Both the overexpression and siRNA transfection were conducted using Lipofectamine 2000 (Invitrogen, CA, USA) according to the manufacturer’s protocol.

### Western blot

The tissues or cultured cells were lysed by radioimmunoprecipitation assay (RIPA; Santa Cruz Biotechnology, Santa Cruz, California, USA) supplemented with a protease inhibitor and phosphatase inhibitor cocktail (Roche, Shanghai, China). Then, a bicinchoninic acid protein assay kit (Biosharp, Shanghai, China) was used to detect the protein concentration. For electrophoresis, 20 μg of the protein sample was loaded on a 10% sodium dodecyl sulfate–polyacrylamide gel electrophoresis (SDS-PAGE) gel and then transferred into polyvinylidene fluoride membrane (PVDF; Millipore, CA, USA) after electrophoresis. Then, membranes were blocked in Tris-buffered saline Tween 20 (TBST) containing 5% skimmed milk for 1 h. These membranes were incubated with primary antibody overnight at 4°C (SRPK1, ab58002, Abcam; Phospho-ERK (Thr202/Tyr204), #4370, Cell Signaling Technology). After washed with TBST for 3 times, PVDF membranes were further incubated with goat secondary antibody IgG-horseradish peroxidase at room temperature for 1 h. Finally, X-ray film (Kodak, NY, USA) was used to analyze the optical density value of target bands. β-actin was served as an internal standard for normalization. Results of densitometric analysis were measured by ImageJ software.

### CCK-8 assay

Cell proliferation was measured using a Cell Counting Kit-8 (Dojindo, Kumamoto, Japan). After transfection and/or drug pre-treatment, SNU-1 and AGS cells were seeded in 96-well plates at 2 × 10^3^ cells/well in triplicate and cultured for designated time (1, 2, 3, 4 days). At each time points, 10 μl of CCK-8 solution was added to each well and incubated for 4 h. The absorbance was measured at 450 nm using a microplate reader. All experiments were repeated three times.

### Invasion assay

Cell invasion assay was conducted using Transwell chamber (Costar, Corning, NY, USA) with Matrigel (BD, NJ, USA) and polycarbonic membrane (6.5mmin diameter, 8 μm pore size). Briefly, the Transwell membrane was pre-coated with Matrigel solution and incubated at 37°C for 4 h. The transfected cells were resuspended at a density of 5 × 10^5^ cells/mL in serum-free medium and 100 μL was added into the upper chamber. 500 μL of DMEM medium supplemented with 10% FBS was then added to the lower chamber. After incubation for 48 h, cells that had invaded to the lower side of the membrane were fixed and stained. Stained cells were counted under a microscope in five randomly chosen fields and the average number was calculated [37]. All experiments were repeated three times.

### Statistical

All data were analyzed by SPSS 18.0 software and presented as mean ± SD. χ2 test was used to explore the association between SRPK1 expression and clinicopathological variables. Kaplan–Meier curves were constructed and the log-rank test was performed for the analysis of survival data. Multivariate analysis was performed using Cox proportional hazards model, and the hazard ratio (HR) as well as its 95% confidence interval (95% CI) were presented. For cellular experiments, all data were expressed as mean ± SD of three independent experiments performed in triplicate. Statistical significance was conducted with the Student's t-test or One-way ANOVA. P < 0.05 was considered statistically significant.

### Ethical

This study was approved by the Ethic Committee of Renmin Hospital Affiliated to Wuhan University. Written consent was obtained from each patient for research purposes. This study complied with the Helsinki Declaration.

## SUPPLEMENTARY MATERIALS FIGURE


