# Supplementary material for: Serine-arginine protein kinase 1 (SRPK1) is elevated in gastric cancer and plays oncogenic functions
Source: Oncotarget. 2017 Jun 28;8(37):61944–57. doi: 10.18632/oncotarget.18734 (PMC5617477; doi:10.18632/oncotarget.18734)
Supplement: Supplementary file 1 [file oncotarget-08-61944-s001.pdf]

Serine-arginine protein kinase 1 (SRPK1) is elevated in gastric cancer and plays oncogenic functions

SUPPLEMENTARY MATERIALS

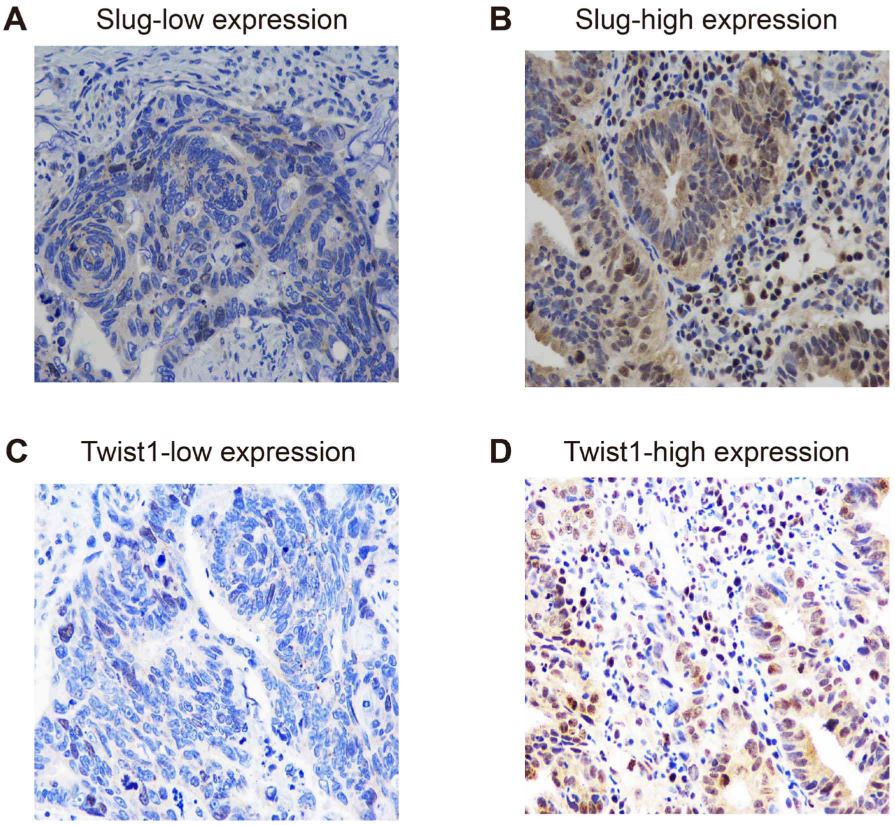

E Correlations between SRPK1 and Slug or Twist1 by chi-square test.

		SRPK1 epxression			
		Low	High	Total	P value
Slug	Low	39	34	73	0.165
	High	36	49	85	
Twist1	Low	45	32	77	0.007*
	High	30	51	81	
	Total	75	83	158	

Supplementary Figure 1: Protein expression of Slug and Twist1 in clinical GC tissues. (A) Representative low expression of Slug. **(B)** Representative high expression of Slug protein, mainly located in the nucleus. **(C)** Representative low expression of Twist1. **(B)** Representative high protein expression of Twist1. **(E)** Correlations between SRPK1 expression with Slug and Twist1.
